# Prenatal PM2.5 exposure and hypertensive disorders of pregnancy: a systematic review and meta-analysis

**DOI:** 10.3389/fpubh.2025.1650913

**Published:** 2025-10-30

**Authors:** Sasa Gao, Han Zhang, Xueling Kang, Xiaoping Cui

**Affiliations:** ^1^Northwest Women's and Children's Hospital, Xi'an, Shaanxi, China; ^2^Shaanxi University of Chinese Medicine, Xianyang, Shaanxi, China

**Keywords:** hypertensive disorders of pregnancy, PM2.5, morbidity, association, pregnancy

## Abstract

**Purpose:**

This study endeavors to unravel the association between PM2.5 exposure and hypertensive disorders of pregnancy (HDP) via a comprehensive review of epidemiological studies.

**Methods:**

Pertinent studies investigating the association between PM2.5 exposure and HDP were retrieved from PubMed, Embase, Web of Science, and the Cochrane Library until June 20, 2024. In addition, one article was identified through an updated search on September 1, 2025. Our study utilized the Newcastle-Ottawa Scale (NOS) and the Journal of Biomedical Informatics (JBI) scale for eligible study quality assessment. Statistical analyses were enabled by R 4.3.2 and Stata 15.1.

**Results:**

Fifteen studies were encompassed, involving 78,427 patients. The meta-analysis revealed the following rates and 95% confidence intervals (CIs): preeclampsia (PE) at 3% (2.9–3.7%); gestational hypertension (GH) at 3% (1.9–4.4%); and HDP at 11.2% (2.1–26%). For the entire pregnancy period, analysis showed a positive association between PM2.5 and PE (OR: 1.11, 95% CI: 1.06, 1.15). In different pregnancy periods, analysis revealed a positive association of PM2.5 with PE (OR: 1.02, 95% CI: 1.01, 1.03). At PM2.5 levels in the third quartile (Q3), analysis showed a positive association with PE (OR: 1.12, 95% CI: 1.07, 1.18). Similarly, at PM2.5 levels in the fourth quartile (Q4), the association was significant (OR: 1.18, 95% CI: 1.12, 1.24). For PM2.5 levels in the second quartile (Q2), a positive association with HDP prevalence was observed (OR: 1.11, 95% CI: 1.00, 1.23). Other analyses suggested that PM2.5 is a risk factor for HDP, though our results lacked statistical significance.

**Conclusion:**

Our study indicates that PM2.5 is a significant risk factor for HDP. Due to several limitations, it was anticipated that future large-scale, multicenter, prospective studies will provide further confirmation of these findings.

## 1 Introduction

Hypertensive disorders of pregnancy (HDP), frequently occurring during pregnancy, are characterized by hypertension, edema, proteinuria, convulsions, coma, heart and brain dysfunction, and even maternal and fetal mortality. The American College of Obstetricians and Gynecologists (ACOG) classifies HDP into four sorts by severity of clinical manifestations: chronic hypertension, gestational hypertension (GH), preeclampsia (PE), and chronic hypertension with PE or eclampsia. HDP influences the health of millions of pregnant women worldwide, not only increasing the risk of cardiovascular diseases (CVDs) in mothers but also potentially having long-term effects on fetal growth and development. McNestry et al. found that HDP is associated with a risen occurrence of CVDs like coronary artery disease, myocardial infarction, coronary artery revascularization, and peripheral artery disease (1). Lyu et al. further identified that HDP could be an important cause of perinatal mortality, causing restricted fetal growth, fetal distress in utero, premature delivery, fetal demise, as well as stillbirth (2). However, the exact causes of HDP remain elusive, with age, weight, body mass index, parity, family history, environmental factors, and air pollution all potentially playing critical roles in its development.

In recent years, with the acceleration of urbanization and the worsening environmental pollution, fine particulate matter (PM2.5, particulate matter with a diameter ≤ 2.5 μm) has become a focal point in public health research. Since it is small, PM2.5 can deeply infiltrate the lung and even permeate into the bloodstream, affecting human health through mechanisms like oxidative stress, inflammatory responses, and others, leading to a wide range of health issues including cardiovascular and respiratory diseases (3). Exposure to air pollution elevates the likelihood of HDP (4).

Although previous studies have explored the association of exposure with PM2.5 with HDP, early findings have been inconclusive. Earlier studies have reported inconsistent results. For example, Shen Yanling et al. found that PM2.5 exposure during the second trimester raises the HDP risk by 14%, suggesting that PM2.5 significantly raises the probability of HDP among Chinese (5). Similarly, Meilin Yan et al. observed that PM2.5 exposure during early pregnancy elevates the likelihood of GH and PE, with incidences of 2.6 and 0.7% (6). However, Chen Guo et al. proved an inverse association of air pollution exposure with HDP (7). Additionally, Carole B. Rudra and colleagues found no association of PM2.5 with PE, nor any close association between air pollutants and preterm birth (8). Moreover, some studies, such as those by Xiujuan Su, revealed no association of PM2.5 exposure with the occurrence of HDP during the second trimester (9). The uncertainty may partly arise from variations in study group, sample size, exposure assessment approaches, regional differences in PM concentrations and makeup, climates, and the inability to differentiate between varying HDP severities. Recent clinical evidence including a retrospective cohort study by Yi Sun et al., has demonstrated that PM2.5 notably elevates HDP incidence, particularly for women in early and mid-pregnancy, with living environment and family income also influencing the prevalence of HDP (10).

## 2 Methods

### 2.1 PROSPERO registration

Our study was registered on the PROSPERO website following the PRISMA guidelines, with the registration number CRD42025596527. This systematic review was conducted and reported following the Preferred Reporting Items for Systematic Reviews and Meta-Analyses (PRISMA) guidelines (11).

### 2.2 PECOS statement

Our systematic review utilized the Population, Exposure, Comparison, Outcome, and Study Design (PECO) statement (12).

Inclusion Criteria:

- Population (P): Pregnant women;
- Exposure (E): High concentrations of PM2.5;
- Comparison (C): Low concentrations of PM2.5;
- Outcome (O): (1) Preeclampsia (PE), gestational hypertension (GH), and HDP under PM2.5 exposure; (2) PE, GH, and HDP risks;
- Study Design (S): Cohort, cross-sectional, and case–control studies.

Exclusion Criteria:

(1) Non-pregnant women populations;
(2) Exposure factors other than PM2.5;
(3) Reviews, reports, meta-analyses, animal studies, non-English publications, conference abstracts, and guideline letters;
(4) Studies with unavailable full text.

### 2.3 Literature search strategy

The Embase, PubMed, Web of Science, and Cochrane Library databases were retrieved for eligible literature until June 20, 2024. Furthermore, a literature update was conducted on September 1, 2025. No language or geographic restrictions were applied. Subject terms and free-text words were used and primarily included “Hypertension,” “Pregnancy-Induced,” “Maternal Hypertension,” “Preeclampsia,” and “Particulate Matter 2.5,” along with all their relevant synonyms. Our strategies and results are presented in Supplementary Table S1. In addition, the citations of eligible articles were scrutinized for additional pertinent studies.

### 2.4 Data extraction

Two researchers independently screened the literature according to the predefined inclusion and exclusion criteria. The selected studies were imported into EndNote 20 for duplicate removal. After de-duplication, the titles and abstracts of the rest were reviewed to ostracize ineligible ones. Full texts were subsequently checked to determine final inclusion. When dissents arose, a third researcher was engaged in making the ultimate decision.

Extracted data encompassed country, study type, population source, sample size, age, patient type, incidence, pregnancy duration, number of pregnancies, PM2.5 exposure levels, outcome measures, and quality assessment. The confounding adjustment factors included maternal age, parity, maternal education, maternal occupation, maternal origin, area-specific deprivation index, season of conception, and year of delivery. If dissents arose, a third researcher resolved the issue.

### 2.5 Study quality assessment

Two independent researchers assessed study quality via the Newcastle-Ottawa Scale (NOS) and the Journal of Biomedical Informatics (JBI) scale. NOS evaluates quality with 8 questions across three domains: Selection, Comparability, and Exposure/Outcome. Beside Comparability (maximum score of 2), all other questions have a maximum score of 1. A score of 7–9 indicates high-quality studies, while a score of 4–7 indicates moderate quality. The JBI scale consists of 10 questions, ranging from non-compliant to comprehensive and correct descriptions, scored 0–2. A score exceeding 14 indicates a low risk of bias. After assessment, the two researchers cross-verified results, and in cases of disagreement, with dissents addressed via a third researcher.

### 2.6 Data synthesis and analysis

This meta-analysis sought to quantitatively evaluate the association between exposure to air pollutants and the likelihood of GH across exposure periods. For consistent comparison of effect estimates, relative risks (RRs) along with their corresponding 95% CIs were derived based on a 10 μg/m^3^ increment in PM2.5 levels (13, 14). The standardized effect estimates were computed through the following formula:


$${\mathrm{RR}}_{\text{Standardized}}=e^{\left(\frac{\text{In}({\mathrm{RR}}_{\text{Original}})}{{\text{Increment}}_{\text{Original}}}\times {\text{Increment}}_{\text{standardized}}\right)}$$

Heterogeneity was assessed via Cochran’s *Q* test and Higgins *I*^2^. *p* for heterogeneity <0.10 or *I*^2^ > 50% denoted significant heterogeneity and the use of a random-effects model, otherwise, a fixed-effect model was employed. When substantial heterogeneity existed, sensitivity and subgroup analyses helped to explore its source. Publication bias was examined via funnel plots, with Egger’s and Begg’s tests for statistical bias analysis.

To assess possible publication bias, Egger’s test was executed, and funnel plot asymmetry was examined when there were over five studies. If publication bias was detected, the trim-and-fill approach was applied to correct the pooled RR. Moreover, sensitivity analyses were undertaken by systematically ostracizing individual studies to evaluate result robustness, provided at least five studies were available.

Every statistical analysis was enabled by R 4.3.2 and STATA 15.1. Every *p*-value was two-tailed, with *p* < 0.05 suggesting statistical significance.

## 3 Results

### 3.1 Literature search and characteristics

**Figure 1:**
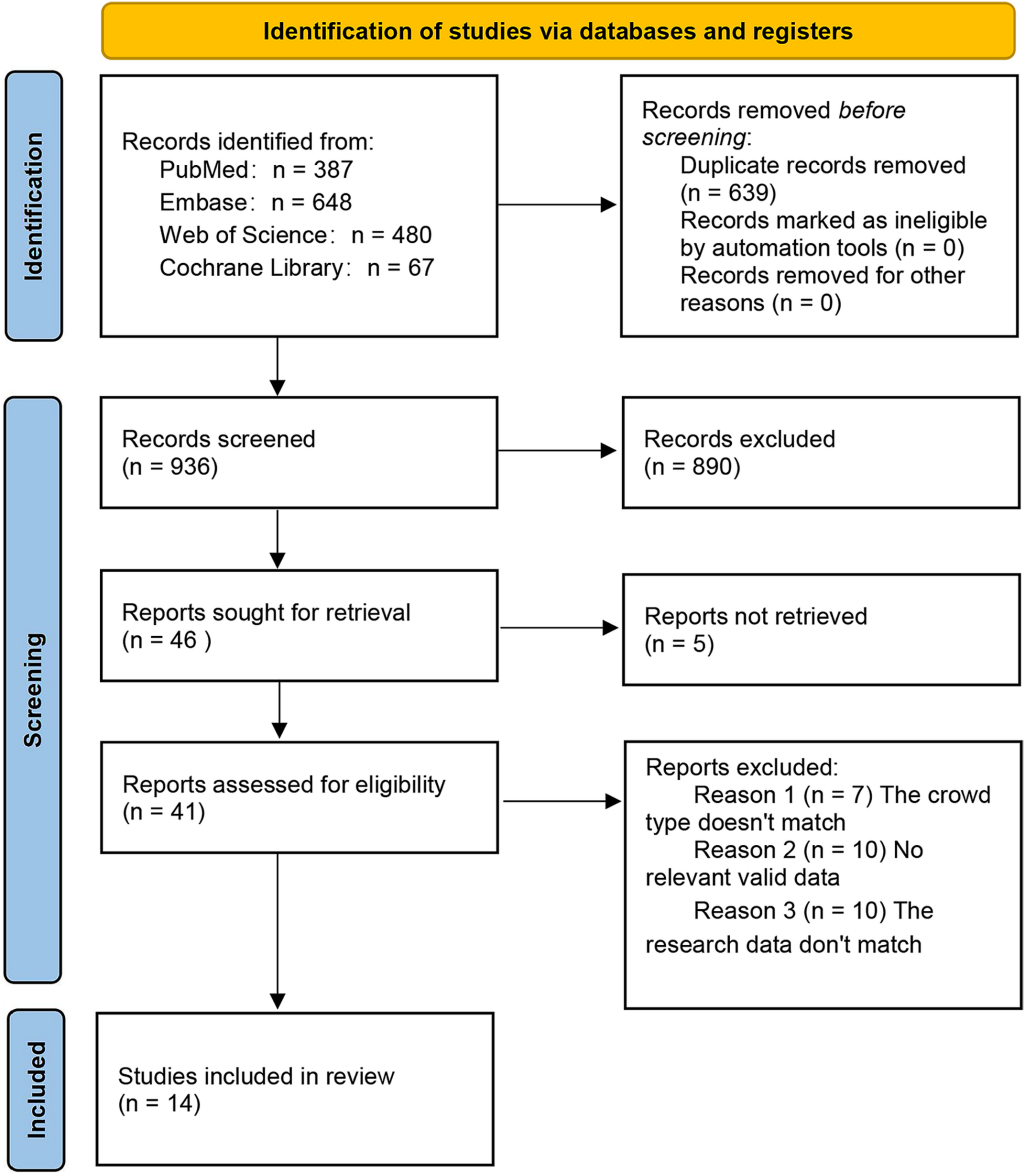
Flowchart of the literature search.

Based on the search strategy, 1,575 potentially relevant records were identified, of which 639 were duplicates. 890 were ineligible in the initial screening. Five records had no full text available. In the second screening, 27 records were excluded: 7 had an irrelevant population type, 10 lacked relevant data, and 10 had mismatched research data. On September 1, 2025, one additional article was identified through the search. The search and screening process is detailed in Figure 1. Ultimately, 15 articles met the inclusion criteria (6, 9, 15–27).

### 3.2 Study population and design

Table 1 displays the baseline characteristics of the 15 eligible studies conducted in Italy (1), China (7), the United States (5), South Korea (1), and Sweden (1) from 2009 to 2025. Among these, 13 were cohort studies, 1 was a cross-sectional study, and 1 was a case–control study. The study populations were from multi-center institutions (11) and single-center institutions (4). The studies covered the period from 1996 to 2022, with a total sample size of 1,361,765 people. The number of patients ranged from 122 to 17,000, all of whom were over 18 years old. The patient types included PE, GH, and HDP. The studies investigated pregnancy in the first, second, and third trimesters, and the full pregnancy period. The PM2.5 concentration was consistent at 10 μg/m^3^. The outcome indicators included the incidence rates of PE, GH, and HDP, as well as the association of PM2.5 with GH, PE, and HDP. Environmental air pollution (PM2.5) levels were categorized into Q1, Q2, Q3, Q4, with Q1 as the reference.

**Table 1:** Characteristics of included studies.

Author	Publication period	Country	Study type	Crowd-source agency	Sample size	Age	Patient		Pregnancy		PM2.5	Outcome indicator	Quality evaluation	Adjustment factors
							Type	Number	Period	Number	Values			
Pedersen (15)	2024	Italy	Cohort study	Multi-center study	130,912	>18	GH	442	First trimester	2,343	7.0 μg/m^3^	1. Incidence of PE and GH	8^a^	Maternal age, parity, maternal education, maternal occupation, maternal origin, area-specific deprivation index, season of conception and year of delivery
							PE	1901	Second trimester			2. Association of PM2.5 with GH and PE		
									Third trimester					
									Full pregnancy					
Yuan (16)	2023	China	Cohort study	Single center study	22,570	>18	HDP	1,520	During the Period From Preconception to Delivery	1,520	10 μg/m^3^	1. HDP incidence 2. Association between PM2.5 and HDP incidence	8^a^	Maternal age (<35 years vs. ≥ 35 years), employment status (employed vs. unemployed), parity (nulliparous vs. multiparous), prepregnancy weight, height, maternal education level (high school or below, junior college, university or above), maternal ethnicity (Han vs. non-Han), first day of last menstrual period, and delivery date.
Yuan (17)	2023	China	Cohort study	Single center study	22,821	29.1 ± 4.1	HDP	1,538	0–13 gestational weeks	1,538	10 μg/m^3^	1. HDP incidence	8^a^	Conception age, employment status, parity, elder gestation, pre-pregnant BMI, maternal education level, maternal ethnicity, temperature, relative humidity, and conceptional year
												2. Association between PM2.5		
									14–20 gestational week					
Li (18)	2023	China	Cohort study	Multi-center study	185,140	30.74 ± 4.02	GH	15,736	First trimester	15,736	10 μg/m^3^	1. Incidence of GH and PE	8^a^	BMI, residence, age, temperature, relative humidity, season, and hospital
							PE		Second trimester			2. Association of PM2.5 with the incidence of GH and PE		
Jiang (19)	2023	China	Cross-sectional study	Multi-center study	67,512	28.70 ± 5.13	GH	1,307	First trimester	2,834	28 ug/m^3^	1. Incidence of GH and PE	8^a^	Maternal age, ethnicity, BMI, insurance status, resident status, education, parity, mode of conception, the season of conception, fetal gender, region, GDP, apparent temperature, and random contribution of the province
							PE	1,527	Second trimester			2. Association of PM2.5 with the incidence of GH and PE		
Yan (6)	2022	China	Cohort study	Multi-center study	3,754	29.6 ± 4.3	GH	96	First trimester	122	10 μg/m^3^	1. Incidence of GH and PE	8^a^	Maternal age (years), maternal body mass index before pregnancy, pre-pregnancy hypertension (yes/no), pre-pregnancy diabetes mellitus (yes/no), weight gains (kg), newborn’s sex (male/female), residential region (mid-west, north, or southeast), and season of conception (spring, summer, autumn, and winter)
							PE	26	Second trimester			2. Association of PM2.5 with GH and PE		
									Third trimester					
Su (9)	2020	China	Cohort study	Single center study	8,776	30.2 ± 3.6	HDP	440	First trimester	440	10 μg/m^3^	1. HDP incidence	8^a^	Maternal age, parity, parental history of chronic diseases, health insurance, sex of fetus, and season of conception
									Second trimester			2. Association of PM2.5 with HDP incidence		
Mandakh (20)	2020	Sweden	Cohort study	Multi-center study	43,688	>18	PE	1,286	Entire pregnancy	1,286	5 μg/m^3^	1. PE incidence	9^a^	Maternal age, body mass index, parity, smoking, diabetes mellitus, gestational diabetes, essential hypertension, gestational hypertension, maternal country of birth, education level, annual household income, fetal sex, and year and season of birth
									1st trimester			2. Association of PM2.5 with PE incidence		
									2nd trimester					
									3rd trimester					
Jia (21)	2020	China	Cohort study	Multi-center study	116,042	>18	PE	2,988	Trimester 1	3,008	NA	1. PE incidence	8^a^	Model, trimester, gravidity, parity, age, education, hospital level, conception and delivery season
									Trimester 2	2,592		2. Association of PM2.5 with PE incidence		
Savitz (22)	2015	USA	Cohort study	Multi-center study	348,585	>18	GH	5,834	First trimester	17,000	10 μg/m^3^	1. Incidence of GH and PE	8^a^	Maternal age, maternal ethnicity, maternal education, Medicaid status, parity, conception year, deprivation index, BMI, BMI2
							PE	11,166	Second trimester			2. Association of PM2.5 with the incidence of PE and GH		
Xu (23)	2014	USA	Cohort study	Multi-center study	22,041	>18	HDP	1,037	First trimester	1,037	1.24 μg/m^3^	1. HDP incidence	8^a^	Maternal age, race, education, marital status, smoking during pregnancy, season of conception, year of conception, prenatal care began and tract median household income
									Second trimester			2. Association of PM2.5 with HDP incidence		
									Full gestational period					
Mobasher (24)	2013	USA	Case–control	Multi-center study	298	27.7 ± 7.4	HDP	136	First trimester	136	7 μg/m^3^	1. HDP incidence	8^a^	Maternal age (continuous), parity, maternal smoking history, exposure to secondhand smoke during pregnancy, and year of conception (before or after 2002)
									Second trimester			2. Association of PM2.5 with HDP incidence		
									Third trimester					
Vinikoor-Imler (25)	2012	USA	Cohort study	Single center study	222,775	>18	GH	12,085	First trimester	12,085	2.24 mg/m^3^	1. GH incidence	8^a^	Maternal age, education, race/ethnicity, marital status, parity and smoking during pregnancy
									Second trimester			2. Association of PM2.5 with GH incidence		
									Third trimester					
Wu (26)	2009	USA	Cohort study	Multi-center study	81,186	30.0 ± 6.2	PE	2,442	Entire pregnancy First trimester	2,442	1.35 μg/m^3^	1. PE incidence	8^a^	Maternal age, maternal race/ethnicity, parity, prenatal care insurance type, poverty, and season of conception
									Second trimester Third trimester			2. Association of PM2.5 with PE incidence		

^a^Quality was evaluated by NOS scale; ^b^Quality evaluation was carried out through JBI scale.

### 3.3 Quality assessment

The study quality was rated through the NOS scale for cohort and case–control studies and the JBI scale for cross-sectional ones. 14 scored 8 points, and 1 had 9 points, all classified as high-quality studies.

### 3.4 Outcome definition

#### 3.4.1 Rate analysis

**Figure 2:**
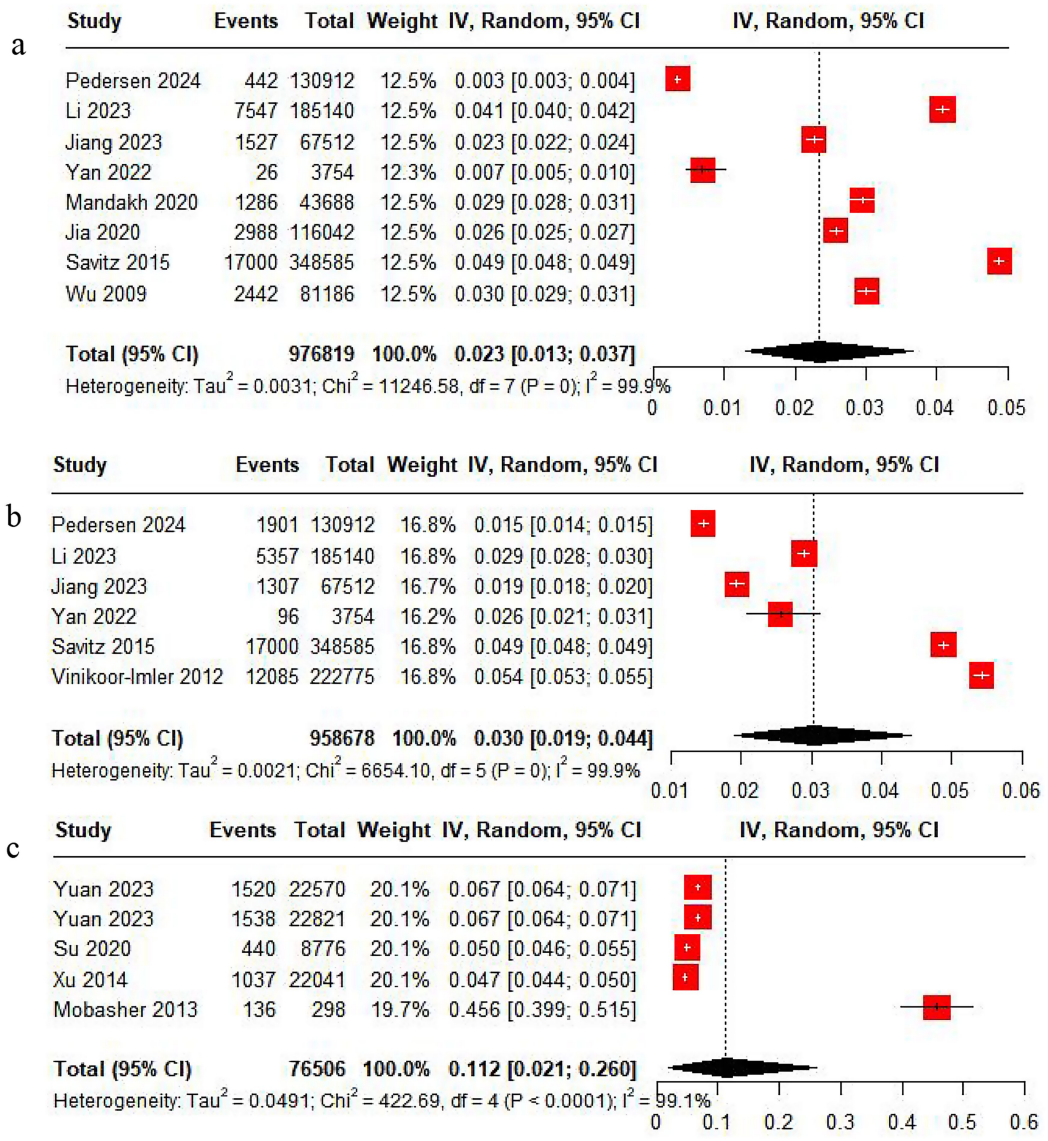
Rate analysis forest map. **(a)** PE rate analysis. **(b)** GH rate analysis. **(c)** HDP rate analysis.

Eight studies reported the incidence of PE. The pooled analysis displayed significant heterogeneity (*I*^2^ = 99.9%), so a random-effects model was leveraged. The incidence of PE was 2.3%, with a 95% CI ranging from 1.3 to 3.7% (Figure 2).

Six studies reported the incidence of GH. The pooled analysis showed significant heterogeneity (*I*^2^ = 99.9%), so a random-effects model was applied. The incidence of GH was 3%, with a 95% CI from 1.9 to 4.4% (Figure 2).

Five studies reported the incidence of HDP. With significant heterogeneity (*I*^2^ = 99.9%) in the pooled analysis, a random-effects model was adopted. The incidence of HDP was 11.2%, with a 95% CI ranging from 2.1 to 26% (Figure 2).

Subgroup analyses by country and sampling year were performed, but neither could help to explain the heterogeneity in PE, GH, and HDP rates (Supplementary Figures S1–S3).

#### 3.4.2 Association analysis

**Figure 3:**
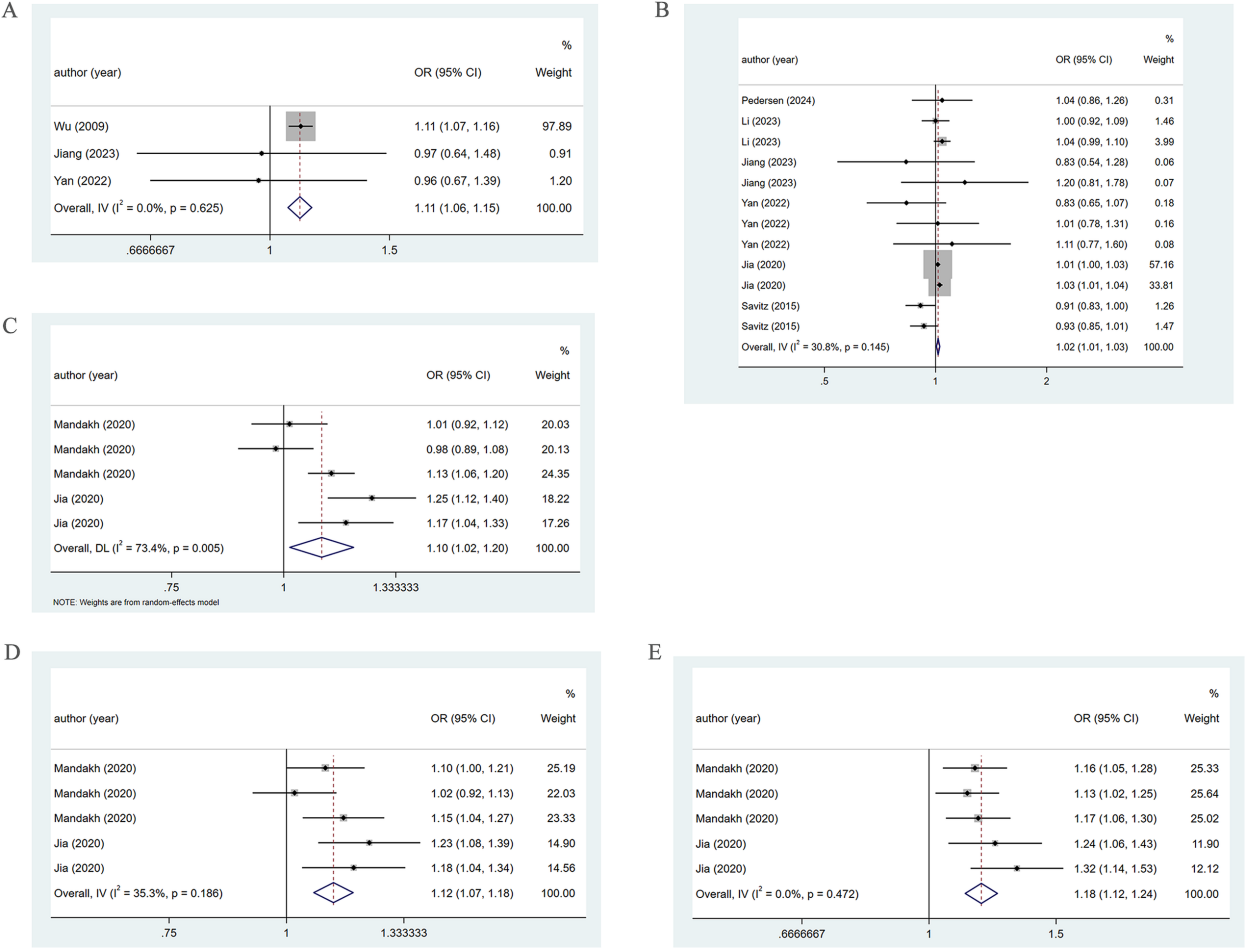
Association between PM2.5 and PE incidence. **(A)** Association between PM2.5 and PE incidence throughout pregnancy. **(B)** According to different pregnancy, PM2.5 and PE incidence association. **(C)** When the PM2.5 concentration level is Q2, the association between PM2.5 and PE incidence. **(D)** When the PM2.5 concentration level is Q3, the association between PM2.5 and PE incidence. **(E)** When the PM2.5 concentration level is Q4, the association between PM2.5 and PE incidence.

Three studies reported the association of PM2.5 with the incidence of PE. The pooled analysis revealed low heterogeneity (*I*^2^ = 0.0%), thus a fixed-effects model was utilized. The analysis indicated a positive association between PM2.5 and the incidence of PE (OR = 1.11, 95% CI: 1.06–1.15) (Figure 3). Five studies reported the association of PM2.5 with PE across trimesters. Low heterogeneity (*I*^2^ = 30.8%) led to the use of a fixed-effects model. There was a positive association between PM2.5 and PE in the first and second trimesters (OR = 1.02, 95% CI: 1.01–1.03) (Figure 3).

When the PM2.5 concentration was at the Q2 level, the pooled analysis showed significant heterogeneity (*I*^2^ = 73.4%), so a random-effects model was used. A positive association of PM2.5 with PE (OR = 1.10, 95% CI: 1.02–1.20) was noted, as shown in Figure 3. At the Q3 level, low heterogeneity (*I*^2^ = 35.3%) led to the adoption of a fixed-effects model. A positive association of PM2.5 with PE (OR = 1.12, 95% CI: 1.07–1.18) was found (Figure 3). At the Q4 level, the pooled analysis showed low heterogeneity (*I*^2^ = 0.0%). Therefore, a fixed-effects model was adopted. The results demonstrated a positive association between PM2.5 and PE (OR = 1.18, 95% CI: 1.12–1.24) (Figure 3). The specific details are presented in Table 1.

**Figure 4:**
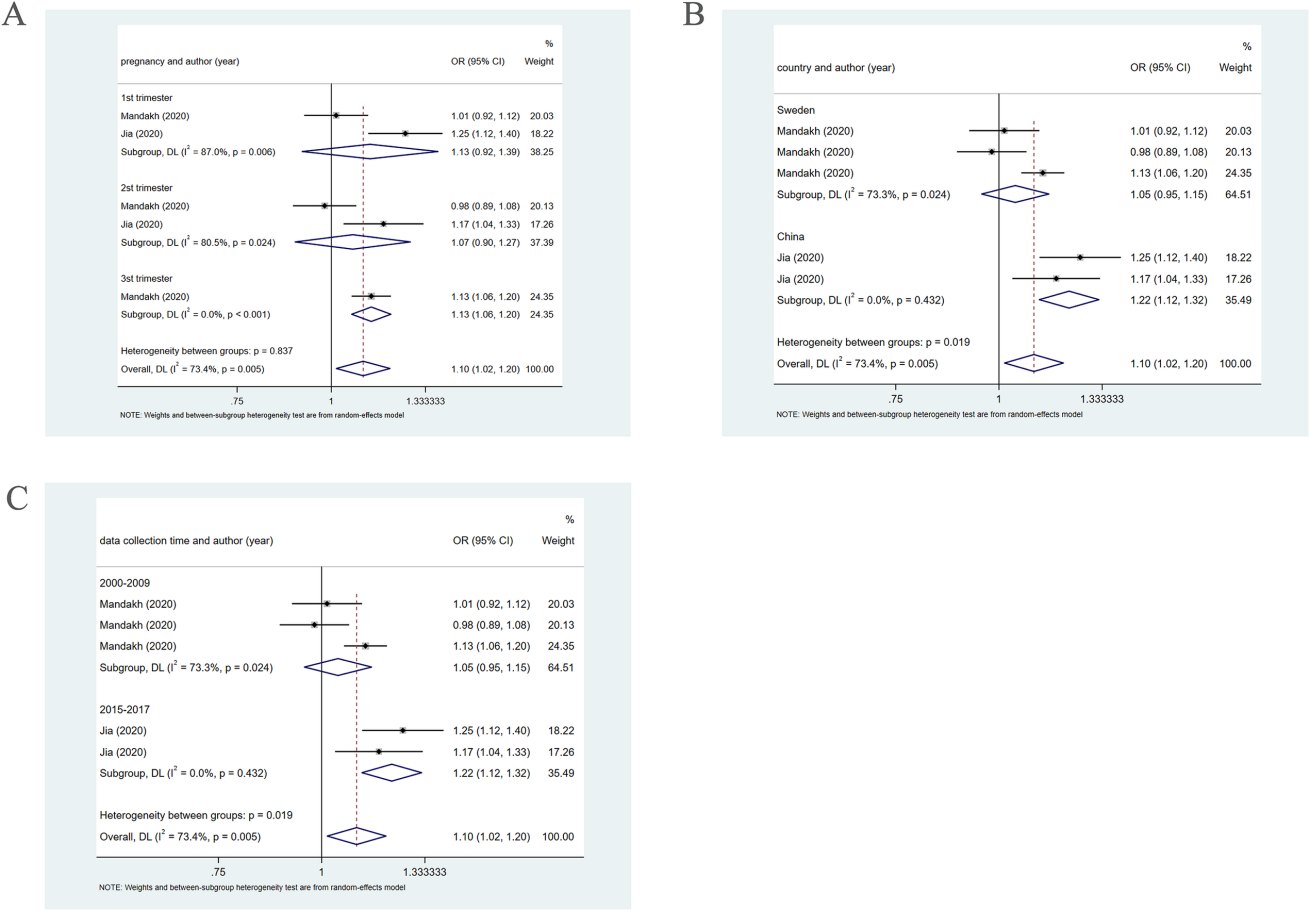
When PM2.5 concentration level is Q2, subgroup analysis. **(A)** Subgroup analysis was performed according to pregnancy. **(B)** Subgroup analysis was performed according to country. **(C)** Subgroup analysis was performed according to sample collection time.

Further subgroup analysis based on pregnancy trimester at the Q2 level employing a random-effects model, revealed the association of PM2.5 exposure in the first and second trimesters with the occurrence of PE (OR = 1.13, 95% CI: 0.92–1.39; OR = 1.07, 95% CI: 0.90–1.27). However, in the third trimester, a positive association of PM2.5 with the prevalence of PE was revealed (OR = 1.13, 95% CI: 1.06–1.20) (Figure 4).

Further subgroup analysis based on country, using a random-effects model, revealed that in Sweden, PM2.5 did not notably correlate with PE (OR = 1.05, 95% CI: 0.95–1.15), whereas in China, PM2.5 positively bore on the incidence of PE (OR = 1.22, 95% CI: 1.12–1.32), as presented in Figure 4. In addition, subgroup analysis based on the year of sample collection, using a random-effects model, revealed that between 2000 and 2009, PM2.5 was not significantly associated with the incidence of PE (OR = 1.05, 95% CI: 0.95–1.15). However, between 2015 and 2017, PM2.5 was positively associated with the incidence of PE (OR = 1.22, 95% CI: 1.12–1.32) (Figure 4), which may be attributable to differences in population characteristics across periods.

**Figure 5:**
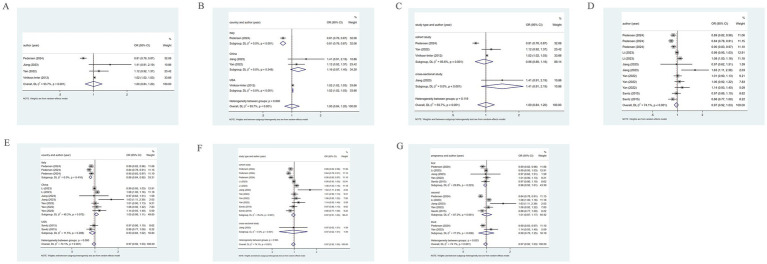
Association between PM2.5 and GH incidence. **(A)** Association between PM2.5 and GH incidence throughout pregnancy; **(B)** Subgroup analysis was performed according to different countries, PM2.5 and GH incidence association; **(C)** Subgroup analysis was performed according to different types of research, PM2.5 and GH incidence association. **(D)** According to different pregnancies, PM2.5 and GH incidence association; **(E)** Subgroup analysis was performed according to different countries, the association between PM2.5 and GH incidence; **(F)** Subgroup analysis was performed according to types of research, the association between PM2.5 and GH incidence; **(G)** Subgroup analysis was performed according to the first, second and third trimester, the association between PM2.5 and GH incidence.

Four studies unveiled the association between PM2.5 and GH. The pooled analysis revealed significant heterogeneity (*I*^2^ = 93.7%). Therefore, a random-effects model was leveraged. PM2.5 was not significantly associated with the incidence of GH (OR = 1.00, 95% CI: 0.84–1.20) (Figure 5). Subgroup analysis based on country, using a random-effects model, revealed that in Italy, PM2.5 did not markedly correlate with GH (OR = 0.81, 95% CI: 0.76–0.87). In China, PM2.5 was not significantly associated with the incidence of GH (OR = 1.16, 95% CI: 0.97–1.40), while in the United States, PM2.5 may be positively connected with the prevalence of GH (OR = 1.02, 95% CI: 1.02–1.03) (Figure 5). Moreover, subgroup analysis based on study type utilizing a random-effects model, revealed that in cohort studies, significant heterogeneity was observed (*I*^2^ = 95.6%), and no significant association of PM2.5 with GH was found (OR = 0.96, 95% CI: 0.80–1.16). In cross-sectional studies, with low heterogeneity (*I*^2^ = 0.0%), the association of PM2.5 with GH was not notable (OR = 1.41, 95% CI: 0.91–2.19) (Figure 5).

Five studies examined the association between PM2.5 and the incidence of GH across the first, second, and third trimesters. The pooled analysis revealed significant heterogeneity (*I*^2^ = 74.1%), prompting the use of a random-effects model. PM2.5 was not markedly associated with GH (OR = 0.97, 95% CI: 0.92–1.03), as depicted in Figure 5. Subgroup analysis based on country, utilizing a random-effects model, revealed that in Italy, PM2.5 was a protective factor for GH (OR = 0.88, 95% CI: 0.84–0.92). However, for China and the United States, PM2.5 was not notably connected with GH (OR = 1.05, 95% CI: 0.98–1.11; OR = 0.93, 95% CI: 0.84–1.02) (Figure 5). Further subgroup analysis based on study type, using a random-effects model, demonstrated that in cohort studies and cross-sectional studies, PM2.5 was not significantly related to the incidence of GH (OR = 0.97, 95% CI: 0.91–1.03; OR = 0.97, 95% CI: 0.62–1.51) (Figure 5). Moreover, when examining by trimester, the analysis leveraging a random-effects model suggested that in the first, second, and third trimesters, PM2.5 exposure did not markedly correlate with GH (OR = 0.96, 95% CI: 0.92–1.01; OR = 1.01, 95% CI: 0.87–1.01; OR = 0.99, 95% CI: 0.79–1.25) (Figure 5).

**Figure 6:**
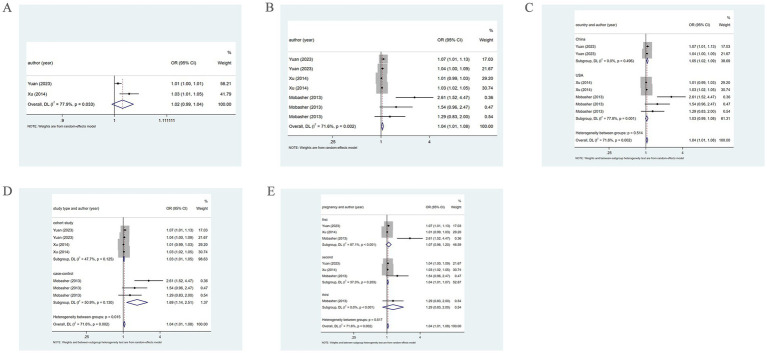
Association between PM2.5 and HDP incidence. **(A)** Association between PM2.5 and HDP incidence throughout pregnancy. **(B)** According to different pregnancy, PM2.5 and HDP incidence association. **(C)** Subgroup analysis was performed according to different countries, PM2.5 and HDP incidence association; **(D)** Subgroup analysis was performed according to different types of research, PM2.5 and HDP incidence association; **(E)** Subgroup analysis was performed according to the first, second and third trimester, the association between PM2.5 and HDP incidence.

Two studies reported the association of PM2.5 with HDP. The pooled analysis revealed significant heterogeneity (*I*^2^ = 77.9%). Therefore, a random-effects model was leveraged. PM2.5 was not notably related to HDP (OR = 1.02, 95% CI: 0.99–1.04), as shown in Figure 6. Three studies reported the association between PM2.5 and HDP across three trimesters. The pooled analysis showed significant heterogeneity (*I*^2^ = 71.6%), prompting the employment of a random-effects model. A positive association of PM2.5 with HDP was demonstrated (OR = 1.04, 95% CI: 1.01–1.08) (Figure 6).

Subgroup analysis based on country, using a random-effects model, revealed that in China, PM2.5 positively correlated with HDP (OR = 1.05, 95% CI: 1.02–1.09). However, in the United States, there was no marked association of PM2.5 with HDP (OR = 1.03, 95% CI: 0.99–1.08), as shown in Figure 6. Further subgroup analysis based on study type revealed that in cohort and case–control studies, PM2.5 was positively associated with HDP (OR = 1.03, 95% CI: 1.01–1.05; OR = 1.69, 95% CI: 1.14–2.51), as shown in Figure 6. Additionally, subgroup analysis by trimester indicated that in the second trimester, PM2.5 positively bore on the incidence of HDP (OR = 1.04, 95% CI: 1.01–1.07). However, in the first and third trimesters, PM2.5 was not significantly related to HDP (OR = 1.07, 95% CI: 0.96–1.20; OR = 1.29, 95% CI: 0.83–2.00) (Figure 6).

**Figure 7:**
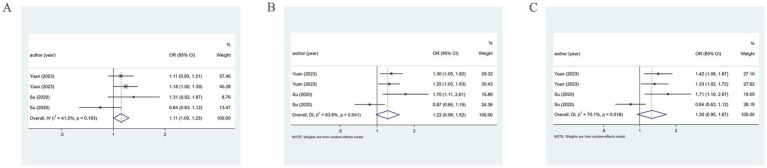
Association between PM2.5 and HDP incidence. **(A)** When the PM2.5 concentration level is Q2, PM2.5 and HDP incidence association. **(B)** When the PM2.5 concentration level is Q3, PM2.5 and HDP incidence association. **(C)** When the PM2.5 concentration level is Q4, PM2.5 and HDP incidence association.

When the PM2.5 concentration level was at Q2, the pooled analysis revealed low heterogeneity (*I*^2^ = 41.5%), so a fixed-effects model was utilized. The results demonstrated a positive association of PM2.5 with the incidence of HDP (OR = 1.11, 95% CI: 1.00–1.23). At the Q3 and Q4 levels, the pooled analysis revealed significant heterogeneity (*I*^2^ = 63.6%; *I*^2^ = 70.1%), so a random-effects model was applied. The results indicated that PM2.5 did not significantly correlate with the incidence of HDP at either concentration level (OR = 1.22, 95% CI: 0.98–1.52; OR = 1.26, 95% CI: 0.95–1.67). As presented in Figure 7. The specific details are provided in Table 1.

When examining by sample collection period at the Q3 and Q4 levels via a random-effects model from 2013 to 2017, PM2.5 exhibited a positive association with HDP (OR = 1.28, 95% CI: 1.10–1.48; OR = 1.37, 95% CI: 1.13–1.66). However, from 2014 to 2015, a significant association between them was not found (OR = 1.19, 95% CI: 0.62–2.29; OR = 1.17, 95% CI: 0.59–1.67). Moreover, subgroup analysis by trimester revealed that in the first trimester, PM2.5 has a positive association with HDP (OR = 1.39, 95% CI: 1.11–1.74; OR = 1.50, 95% CI: 1.18–1.89). However, in the second trimester, no evident connection was found (OR = 1.06, 95% CI: 0.74–1.51; OR = 1.06, 95% CI: 0.68–1.66).

#### 3.4.3 Sensitivity analysis

**Figure 8:**
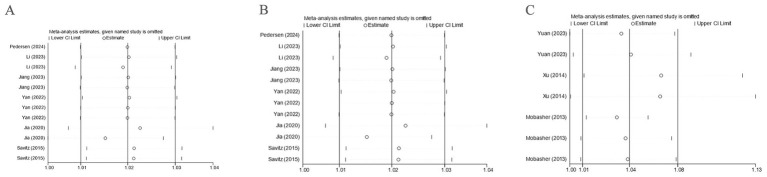
Sensitivity analysis. **(A)** Sensitivity analysis was performed with PE as the outcome; **(B)** Sensitivity analysis was performed with GH as the outcome; **(C)** Sensitivity analysis was performed with HDP as the outcome.

Sensitivity analysis was executed for PE, GH, and HDP. By sequentially removing each study and observing the pooled effect size of the remaining studies, it was found that the differences between the new results and the original outcomes were minimal, indicating that the results were stable. The study by Lee (27) was excluded. Detailed results are provided in Figure 8.

#### 3.4.4 Publication bias analysis

For the studies with PE as the outcome, publication bias was detected via a funnel plot, which visually demonstrated potential publication bias. Further, Egger’s test was employed for assessing bias in the funnel plot. The results showed that the *p*-values for the entire pregnancy (*p* = 0.074), different trimesters (*p* = 0.971), PM2.5 level Q2 (*p* = 0.991), and PM2.5 level Q3 (*p* = 0.245) were all greater than 0.05, so there was no marked publication bias. However, when PM2.5 concentration was at Q4, Egger’s test revealed a *p*-value of 0.036, demonstrating the presence of publication bias.

For the studies with GH as the outcome, publication bias was also assessed using a funnel plot, and Egger’s test was applied. The *p*-values for the entire pregnancy (*p* = 0.674) and different trimesters (*p* = 0.516) were both greater than 0.05, suggesting no significant publication bias.

For the studies with HDP as the outcome, publication bias was evaluated based on different trimesters using a funnel plot, and Egger’s test was executed. The *p*-value (*p* = 0.025) was less than 0.05, indicating publication bias. However, when PM2.5 levels were at Q2, Q3, and Q4, the *p*-values (*p* = 0.764, *p* = 0.868, and *p* = 0.681) were all greater than 0.05, suggesting no significant publication bias.

## 4 Discussion

In this study, relevant literature was collected from four major English-language databases and employed a meta-analysis approach. Our study found varying prevalence rates for different outcomes: the prevalence of PE and GH was 3%, while the prevalence of HDP was 11.2%. The association of PM2.5 exposure with the incidence of different outcomes also varied. For example, when PM2.5 concentration was at Q2, a prominent association was not observed between PM2.5 and PE incidence in Sweden, whereas in China, a positive association was noted. In both Italy and China, no marked association was noted between PM2.5 and GH incidence, but in America, a positive association was noted. In China, PM2.5 exposure exhibited a positive connection with HDP incidence, while in the United States, no significant association was observed.

Our study further corroborates previous findings that exposure to PM2.5 significantly elevated the likelihood of PE (9), with our results showing a significant positive association of PM2.5 with PE incidence. The underlying mechanisms may involve the entry of air pollutants into the bloodstream, inducing systemic oxidative stress, the release of inflammatory factors, endothelial dysfunction, and vasoconstriction. Other studies have also shown that air pollutants give rise to insulin resistance, causing hyperinsulinemia, reduced nitric oxide synthesis, lipid metabolism abnormalities, and the altered synthesis of prostaglandin E2, which increases peripheral vascular resistance and consequently raises blood pressure (21), ultimately resulting in the development of pregnancy-induced hypertension. Since the newly added study by Lee (27) involved a study population distinct from the others, consisting exclusively of patients with preeclampsia rather than healthy pregnant women, inclusion of its data in the analysis would compromise the stability of the pooled results. Therefore, this study was excluded during the sensitivity analysis.

Yi Sun et al. found that the most influential time windows for PM2.5 exposure related to HDP were early and mid-pregnancy, with factors influencing HDP risk also varying according to different countries and economic income levels (10). Our study found that when PM2.5 levels were at Q2, a notable positive association with the incidence of HDP was observed. However, in other cases, the results showed no statistically significant association. Given the high heterogeneity, subgroup analyses were executed based on factors such as country, pregnancy trimester, and study type. These analyses revealed a significant positive association between PM2.5 exposure and HDP in Chinese populations, during mid- and late-pregnancy, and in cohort studies. Furthermore, when PM2.5 levels were at Q3 and Q4, subgroup analyses indicated a notable positive association of PM2.5 with HDP during early pregnancy and in samples collected between 2013 and 2017. Additionally, Wei Bai et al.’s systematic review and meta-analysis, based on cohort studies, demonstrated that PM2.5 exposure significantly raises the probability of HDP throughout pregnancy, particularly during early pregnancy (28) Moreover, Mengqi Sun et al. found that each 10 μg/m^3^ elevation in PM2.5 exposure, the likelihood of HDP in pregnant women increased, especially during early and late pregnancy (29). Variations in factors such as the country of the studied population, pregnancy trimester, research design, sample collection methods, and PM2.5 assessment criteria may contribute to the differences in results. Differences in dietary structures, lifestyle habits, and genetic factors between domestic and international populations could also lead to varied responses to PM2.5 exposure. For example, while Western populations tend to have high-protein, low-carbohydrate diets, Chinese populations typically consume diets higher in carbohydrates and lower in protein. High-quality, high-protein diets can enhance immune function and potentially reduce disease risk. In cases where PM2.5 levels are low, the risk of HDP may increase in the Chinese population but have no significant impact on American pregnant women. During mid-pregnancy and late-pregnancy, as the physical burden increases and immunity declines, the risk of HDP rises. However, high levels of PM2.5 exposure possibly increase the risk of HDP even in early pregnancy.

Additionally, our study analyzed the group with GH as the outcome and found no statistically significant association of PM2.5 with the incidence of GH. This differs slightly from previous studies. For example, Cheng Li et al. demonstrated that PM2.5 was significantly related to the incidence of GH during early pregnancy, especially for women who became pregnant through assisted reproductive technologies. GH risk brought by PM2.5 was even higher in the later stages of pregnancy (18). Jiang Wen et al., through a multicenter study, found that PM2.5 was the main air pollutant responsible for the development of GH (19). However, Yi Sun et al. found no statistically significant association of PM2.5 with GH^10^, which is consistent with our findings. Factors that may explain this discrepancy include PM2.5 assessment methods, geographic location, and economic environment. For example, differences in the evaluators of the study results may lead to inconsistent statistical methods for exposure factors. Furthermore, the life areas of the study participants may differ, which could lead to discrepancies in the quantification of PM2.5 exposure. In addition, PM2.5 may have varying mechanisms of impact on different degrees of HDP.

The differing associations between air pollution and HDP are possibly related to the different mechanisms through which PM2.5 affects varying degrees of HDP. HDP is a relatively mild form of HDP, where the impact of PM2.5 exposure is greatest in early pregnancy. One study observed that animals exposed to PM2.5 developed a persistent state of endometritis, and pathological changes in the placenta and vascular damage were also observed in mice after exposure (9). Studies have confirmed that PM2.5 upregulates cytochrome P-450 and induces stress response enzymes (10). P-450 liver enzyme induction pathway leads to the rapid clearance of vasoconstrictor cytokines from the system. Therefore, in patients with mild HDP, PM2.5 may cause vascular dilation by inducing the breakdown of vasoconstrictor factors, thus not increasing the risk of GH. This is consistent with our findings. Additionally, the placenta is a key factor in the onset of PE. Damage to trophoblast invasion and the release of placental vascular active substances are thought to cause vasoconstriction and placental hypoxia, which are believed to be mechanisms leading to the onset of PE (30). PM2.5 exposure is associated with endothelial dysfunction (31). Endothelial dysfunction is a key marker of PE. Clinical and experimental evidence proved that systemic endothelial proliferation in the body, kidneys, brain, and liver circulation reduces the production of vasodilators derived from the endothelium like nitric oxide, prostacyclin, as well as hyperpolarizing factors while increasing vasoconstrictors like endothelin-1 and thromboxane A2, elevating vasoconstriction and high blood pressure, which are signs of PE (30). Due to PM2.5 exposure, both early and late PE present endothelial dysfunction, which is also verified by our study results.

Our study results suggest that PM2.5 markedly raises PE risk, and under certain conditions, also significantly elevates the risk of HDP. For instance, in the Chinese population, PM2.5 is associated with HDP, whereas among Americans, PM2.5 is not related to the incidence of HDP. Additionally, our results demonstrate that the association between PM2.5 and GH is not statistically significant. Therefore, it was recommended that pregnant women and those planning pregnancy carefully select their living environments, avoiding exposure to harmful air pollution, especially PM2.5. Measures such as wearing masks when outdoors, regularly cleaning living spaces, and using air purifiers can help reduce exposure to harmful substances while protecting fetal development and growth, thereby decreasing the incidence of pregnancy complications.

This study serves as an update to existing literature, further clarifying the prevalence of subtypes of pregnancy-related hypertensive disorders and the associations of PM2.5 with these disorders. Moreover, additional analyses were carried out from the perspectives of country of origin, pregnancy stage, and study type, making this data more applicable to the current state of research. However, there are limitations: (1) A limited number of studies were included, particularly those examining the association between PM2.5 and HDP and GH, and further research is needed to explore this association, thereby strengthening the reliability of the analytical results. (2) The studies selected for this research were published in English, which may introduce publication bias. Moreover, most of the included studies originated from China, which may introduce regional bias and affect the generalizability of the findings. Future investigations are therefore encouraged to be conducted across more diverse regions. (3) Different countries may adopt different statistical standards for disease definitions and PM2.5 assessment criteria, or employ different monitoring methods for PM2.5, which could influence the findings. Therefore, our study results should be interpreted with caution, and further standardized research with more detailed data is warranted. (4) Although the included studies accounted for multivariate analyses and thus provided relatively reliable results, the confounding factors adjusted for in each study were not entirely consistent. Only a subset of studies accounted for individual-level and other potential confounding factors, which may have exerted a potential impact on the findings. Accordingly, the results should be interpreted cautiously, and future research is expected to incorporate more comprehensive and systematic analyses of these factors. In the present review, the most frequently adjusted confounders were maternal age, parity, body mass index, and season, while some studies additionally adjusted for maternal race/ethnicity and smoking history. Future studies should systematically summarize and standardize these adjustments in order to better delineate the independent effects of PM2.5 on hypertensive disorders of pregnancy. (5) The subgroup analyses in this study were exploratory in nature and involved limited samples. Therefore, corrections for multiple comparisons were not applied. The corresponding results should thus be regarded as hypothesis-generating and require validation in future, larger-scale investigations.

## 5 Conclusion

Air pollutant PM2.5 significantly increases the risk of PE. Under certain conditions, it also raises the risk of HDP and GH. Factors influencing the stability of these results may include geographic location, study type, and PM2.5 assessment standards. It is anticipated that future large-scale, multicenter studies will provide unified disease definitions, PM2.5 assessment standards, and reliable data to guide clinical practice and protect the physical and mental health of pregnant women.

## Data Availability

The original contributions presented in the study are included in the article/Supplementary material, further inquiries can be directed to the corresponding author.

## Supplementary material

SUPPLEMENTARY FIGURE S1 Subgroup analysis of GH rate. **(A)** Based on different countries; **(B)** Based on sampling year.

SUPPLEMENTARY FIGURE S2 Subgroup analysis of PE rate. **(A)** Based on different countries; **(B)** Based on sampling year.

SUPPLEMENTARY FIGURE S3 Subgroup analysis of HDP rate. **(A)** Based on different countries; **(B)** Based on sampling year.
