# Supplementary figures and images for: Prenatal PM2.5 exposure and hypertensive disorders of pregnancy: a systematic review and meta-analysis
Source: Front Public Health. 2025 Oct 30;13:1650913. doi: 10.3389/fpubh.2025.1650913 (PMC12611668; doi:10.3389/fpubh.2025.1650913)

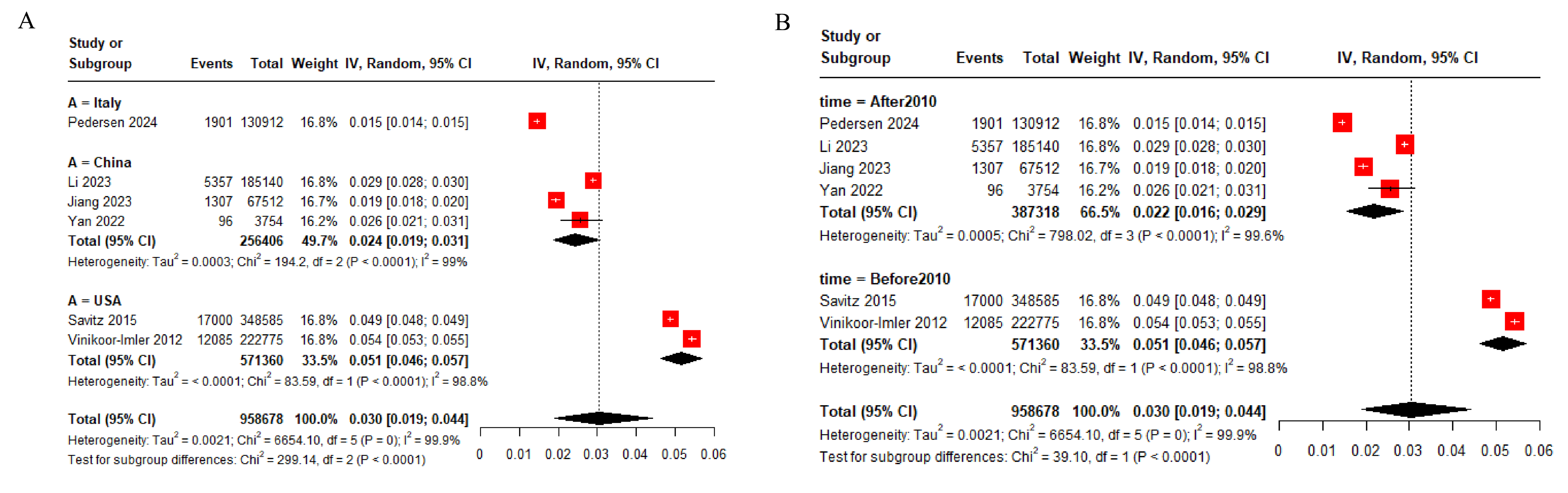

Supplement: SUPPLEMENTARY FIGURE S1 — Subgroup analysis of GH rate. (A) Based on different countries; (B) Based on sampling year. [file Image_1.tif]

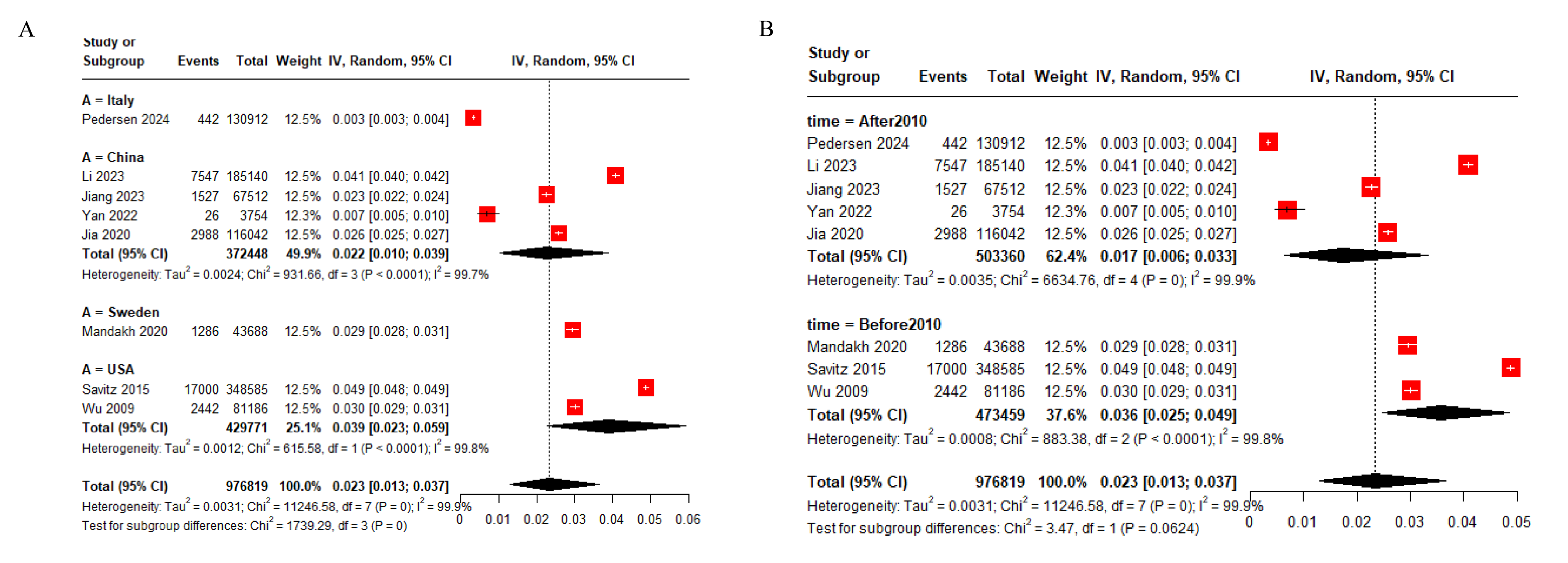

Supplement: SUPPLEMENTARY FIGURE S2 — Subgroup analysis of PE rate. (A) Based on different countries; (B) Based on sampling year. [file Image_2.tif]

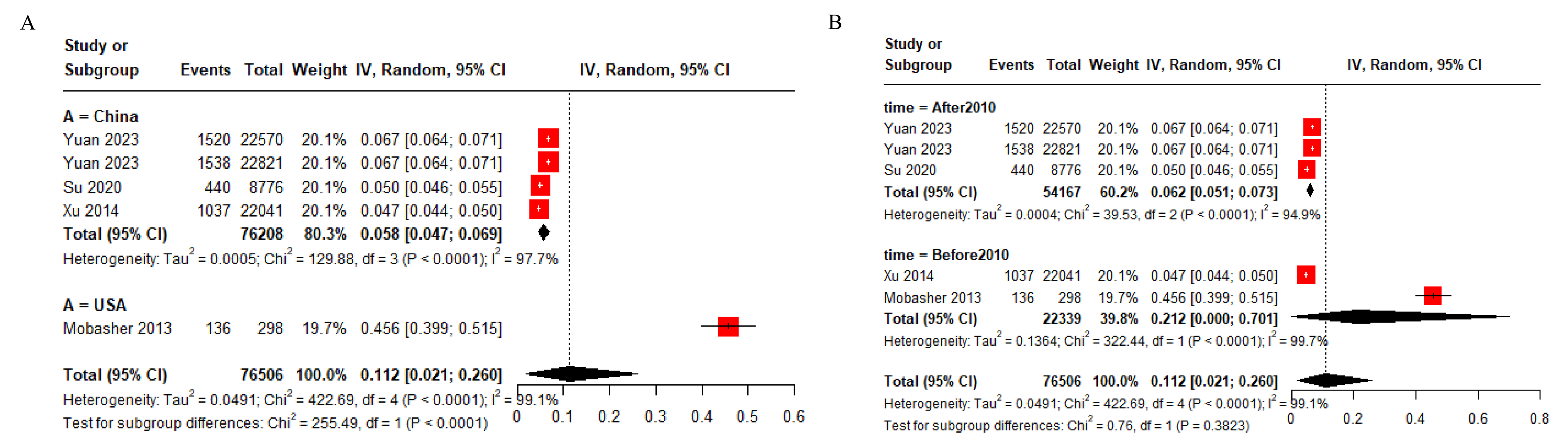

Supplement: SUPPLEMENTARY FIGURE S3 — Subgroup analysis of HDP rate. (A) Based on different countries; (B) Based on sampling year. [file Image_3.tif]
