# Supplementary material for: Prenatal PM2.5 exposure and hypertensive disorders of pregnancy: a systematic review and meta-analysis
Source: Front Public Health. 2025 Oct 30;13:1650913. doi: 10.3389/fpubh.2025.1650913 (PMC12611668; doi:10.3389/fpubh.2025.1650913)
Supplement: Supplementary file 4 [file Table_1.docx]

**Table S1: Search strategies**

**Pubmed**

**#1** (Hypertension, Pregnancy-Induced[MeSH Terms]) OR (Pre-Eclampsia[MeSH Terms])

**#2** (((((((((((((((((((((((((((((((((((((((((((((((((((((((((((((((((((((Hypertension, Pregnancy-Induced[Title/Abstract]) OR (Hypertension, Pregnancy Induced[Title/Abstract])) OR (Pregnancy-Induced Hypertension[Title/Abstract])) OR (Gestational Hypertension[Title/Abstract])) OR (Hypertension, Gestational[Title/Abstract])) OR (Pregnancy Induced Hypertension[Title/Abstract])) OR (Hypertensions, Pregnancy Induced[Title/Abstract])) OR (Induced Hypertension, Pregnancy[Title/Abstract])) OR (Induced Hypertensions, Pregnancy[Title/Abstract])) OR (Transient Hypertension, Pregnancy[Title/Abstract])) OR (Hypertension, Pregnancy Transient[Title/Abstract])) OR (Pregnancy Transient Hypertension[Title/Abstract])) OR (hypertensive disorders of pregnancy[Title/Abstract])) OR (HDP[Title/Abstract])) OR (maternal hypertension[Title/Abstract])) OR (hypertension during pregnancy[Title/Abstract])) OR (hypertension in pregnancyhypertension induced by pregnancy[Title/Abstract])) OR (hypertension, maternal[Title/Abstract])) OR (hypertension, pregnancy induced[Title/Abstract])) OR (hypertension, pregnancy-induced[Title/Abstract])) OR (hypertensive disorder of pregnancy[Title/Abstract])) OR (PIH[Title/Abstract])) OR (pregnancy induced hypertension[Title/Abstract])) OR (pregnancy associated hypertension[Title/Abstract])) OR (pregnancy hypertension[Title/Abstract])) OR (pregnancy induced hypertension[Title/Abstract])) OR (pregnancy-induced hypertension[Title/Abstract])) OR (Pre-Eclampsia[Title/Abstract])) OR (Preeclampsia[Title/Abstract])) OR (pre eclampsia[Title/Abstract])) OR (pre-eclampsia[Title/Abstract])) OR (Preeclamptic[Title/Abstract])) OR (proteinuric hypertension of pregnancy[Title/Abstract])) OR (preeclampsia[Title/Abstract])) OR (Pre Eclampsia[Title/Abstract])) OR (Preeclampsia[Title/Abstract])) OR (Pregnancy Toxemias[Title/Abstract])) OR (Pregnancy Toxemia[Title/Abstract])) OR (Toxemia, Pregnancy[Title/Abstract])) OR (Edema-Proteinuria-Hypertension Gestosis[Title/Abstract])) OR (Edema Proteinuria Hypertension Gestosis[Title/Abstract])) OR (Gestosis, Edema-Proteinuria-Hypertension[Title/Abstract])) OR (Hypertension-Edema-Proteinuria Gestosis[Title/Abstract])) OR (Gestosis, Hypertension-Edema-Proteinuria[Title/Abstract])) OR (Hypertension Edema Proteinuria Gestosis[Title/Abstract])) OR (Proteinuria-Edema-Hypertension Gestosis[Title/Abstract])) OR (Gestosis, Proteinuria-Edema-Hypertension[Title/Abstract])) OR (Proteinuria Edema Hypertension Gestosis[Title/Abstract])) OR (EPH Complex[Title/Abstract])) OR (EPH Toxemias[Title/Abstract])) OR (EPH Toxemia[Title/Abstract])) OR (Toxemia, EPH[Title/Abstract])) OR (Toxemias, EPH[Title/Abstract])) OR (EPH Gestosis[Title/Abstract])) OR (Gestosis, EPH[Title/Abstract])) OR (Toxemias, Pregnancy[Title/Abstract])) OR (Toxemia Of Pregnancy[Title/Abstract])) OR (Of Pregnancies, Toxemia[Title/Abstract])) OR (Of Pregnancy, Toxemia[Title/Abstract])) OR (Pregnancies, Toxemia Of[Title/Abstract])) OR (Pregnancy, Toxemia Of[Title/Abstract])) OR (Toxemia Of Pregnancies[Title/Abstract])) OR (Preeclampsia Eclampsia 1[Title/Abstract])) OR (1, Preeclampsia Eclampsia[Title/Abstract])) OR (1s, Preeclampsia Eclampsia[Title/Abstract])) OR (Eclampsia 1, Preeclampsia[Title/Abstract])) OR (Eclampsia 1s, Preeclampsia[Title/Abstract])) OR (Preeclampsia Eclampsia 1s[Title/Abstract])) OR (preeclampsia[Title/Abstract])) OR (Preeclamptic[Title/Abstract])

**#3** ((maternal[Title/Abstract]) OR (pregnancy[Title/Abstract])) OR (pregnant[Title/Abstract])

**#4** ((pre-eclampsia[Title/Abstract]) OR (hypertension[Title/Abstract])) OR (blood pressure[Title/Abstract])

**#5** #3 AND #4

**#6** #1 OR #2 OR #5

**#7** ((Air Pollution[MeSH Terms]) OR (Air Pollutants[MeSH Terms])) OR (particulate matter[MeSH Terms])

**#8** (((((((((((((((((((((((((((((((((((((((((((((((((((((((((Air Pollution[Title/Abstract]) OR (Air Pollutions[Title/Abstract])) OR (Pollution, Air[Title/Abstract])) OR (Air Quality[Title/Abstract])) OR (particulate matter 2.5[Title/Abstract])) OR (fine particulate matter[Title/Abstract])) OR (inhalable fine particles[Title/Abstract])) OR (inhalable particles PM2.5[Title/Abstract])) OR (particulate matter 2.5 micrometers[Title/Abstract])) OR (particulate matter 2.5[Title/Abstract])) OR (air pollution[Title/Abstract])) OR (aerial pollution[Title/Abstract])) OR (air contamination[Title/Abstract])) OR (air pollutioning[Title/Abstract])) OR (air-borne pollution[Title/Abstract])) OR (airborne pollution[Title/Abstract])) OR (atmosphere pollution[Title/Abstract])) OR (atmospheric pollution[Title/Abstract])) OR (polluted air[Title/Abstract])) OR (polluted atmosphere[Title/Abstract])) OR (pollution, air[Title/Abstract])) OR (air pollution[Title/Abstract])) OR (Air Pollutant[Title/Abstract])) OR (air pollutants[Title/Abstract])) OR (air pollutants, occupational[Title/Abstract])) OR (pollutant, air[Title/Abstract])) OR (air pollutant[Title/Abstract])) OR (particulate matter[Title/Abstract])) OR (Air Pollutants[Title/Abstract])) OR (Pollutants, Air[Title/Abstract])) OR (Air Pollutant[Title/Abstract])) OR (Pollutant, Air[Title/Abstract])) OR (Air Pollutants, Environmental[Title/Abstract])) OR (Environmental Pollutants, Air[Title/Abstract])) OR (Air Environmental Pollutants[Title/Abstract])) OR (Pollutants, Air Environmental[Title/Abstract])) OR (Environmental Air Pollutants[Title/Abstract])) OR (Pollutants, Environmental Air[Title/Abstract])) OR (particulate matter[Title/Abstract])) OR (Airborne Particulate Matter[Title/Abstract])) OR (Particulate Matter, Airborne[Title/Abstract])) OR (Ambient Particulate Matter[Title/Abstract])) OR (Particulate Matter, Ambient[Title/Abstract])) OR (Air Pollutants, Particulate[Title/Abstract])) OR (Particulate Air Pollutants[Title/Abstract])) OR (Ultrafine Particulate Matter[Title/Abstract])) OR (Particulate Matter, Ultrafine[Title/Abstract])) OR (Ultrafine Particle[Title/Abstract])) OR (Particle, Ultrafine[Title/Abstract])) OR (Ultrafine Particles[Title/Abstract])) OR (Particles, Ultrafine[Title/Abstract])) OR (Ultrafine Fibers[Title/Abstract])) OR (Ultrafine Fiber[Title/Abstract])) OR (Fiber, Ultrafine[Title/Abstract])) OR (traffic pollution[Title/Abstract])) OR (PM2.5[Title/Abstract])) OR (outdoor pollution[Title/Abstract])) OR (indoor pollution[Title/Abstract])

**#9** #7 OR #8

**#10** #6 AND #9

**Cochrane Library**

**#1** MeSH descriptor: [Hypertension, Pregnancy-Induced] explode all trees

**#2** MeSH descriptor: [Pre-Eclampsia] explode all trees

**#3** (Hypertension, Pregnancy-Induced):ti,ab,kw OR (Hypertension, Pregnancy Induced):ti,ab,kw OR (Pregnancy-Induced Hypertension):ti,ab,kw OR (Gestational Hypertension):ti,ab,kw OR (Hypertension, Gestational):ti,ab,kw

**#4** (Pregnancy Induced Hypertension):ti,ab,kw OR (Hypertensions, Pregnancy Induced):ti,ab,kw OR (Induced Hypertension, Pregnancy):ti,ab,kw OR (Induced Hypertensions, Pregnancy):ti,ab,kw OR (Transient Hypertension, Pregnancy):ti,ab,kw

**#5** (Hypertension, Pregnancy Transient):ti,ab,kw OR (Pregnancy Transient Hypertension):ti,ab,kw OR (hypertensive disorders of pregnancy):ti,ab,kw OR (HDP):ti,ab,kw OR (maternal hypertension):ti,ab,kw

**#6** (hypertension during pregnancy):ti,ab,kw OR (hypertension in pregnancy):ti,ab,kw OR (hypertension induced by pregnancy):ti,ab,kw OR (hypertension, maternal):ti,ab,kw OR (hypertension, pregnancy induced):ti,ab,kw

**#7** (hypertension, pregnancy-induced):ti,ab,kw OR (hypertensive disorder of pregnancy):ti,ab,kw OR (PIH):ti,ab,kw OR (pregnancy induced hypertension):ti,ab,kw OR (pregnancy associated hypertension):ti,ab,kw

**#8** (pregnancy hypertension):ti,ab,kw OR (pregnancy induced hypertension):ti,ab,kw OR (pregnancy-induced hypertension):ti,ab,kw OR (Pre-Eclampsia):ti,ab,kw OR (Preeclampsia):ti,ab,kw

**#9** (pre eclampsia):ti,ab,kw OR (pre-eclampsia):ti,ab,kw OR (Preeclamptic):ti,ab,kw OR (proteinuric hypertension of pregnancy):ti,ab,kw OR (preeclampsia):ti,ab,kw

**#10** (Pre Eclampsia):ti,ab,kw OR (Preeclampsia):ti,ab,kw OR (Pregnancy Toxemias):ti,ab,kw OR (Pregnancy Toxemia):ti,ab,kw OR (Toxemia, Pregnancy):ti,ab,kw

**#11** (Edema-Proteinuria-Hypertension Gestosis):ti,ab,kw OR (Edema Proteinuria Hypertension Gestosis):ti,ab,kw OR (Gestosis, Edema-Proteinuria-Hypertension):ti,ab,kw OR (Hypertension-Edema-Proteinuria Gestosis):ti,ab,kw OR (Gestosis, Hypertension-Edema-Proteinuria):ti,ab,kw

**#12** (Hypertension Edema Proteinuria Gestosis):ti,ab,kw OR (Proteinuria-Edema-Hypertension Gestosis):ti,ab,kw OR (Gestosis, Proteinuria-Edema-Hypertension):ti,ab,kw OR (Proteinuria Edema Hypertension Gestosis):ti,ab,kw OR (EPH Complex):ti,ab,kw

**#13** (EPH Toxemias):ti,ab,kw OR (EPH Toxemia):ti,ab,kw OR (Toxemia, EPH):ti,ab,kw OR (Toxemias, EPH):ti,ab,kw OR (EPH Gestosis):ti,ab,kw

**#14** (Gestosis, EPH):ti,ab,kw OR (Toxemias, Pregnancy):ti,ab,kw OR (Toxemia Of Pregnancy):ti,ab,kw OR (Of Pregnancies, Toxemia):ti,ab,kw OR (Of Pregnancy, Toxemia):ti,ab,kw

**#15** (Pregnancies, Toxemia Of):ti,ab,kw OR (Pregnancy, Toxemia Of):ti,ab,kw OR (Toxemia Of Pregnancies):ti,ab,kw OR (Preeclampsia Eclampsia 1):ti,ab,kw OR (1, Preeclampsia Eclampsia):ti,ab,kw

**#16** (1s, Preeclampsia Eclampsia):ti,ab,kw OR (Eclampsia 1, Preeclampsia):ti,ab,kw OR (Eclampsia 1s, Preeclampsia):ti,ab,kw OR (Preeclampsia Eclampsia 1s):ti,ab,kw OR (preeclampsia):ti,ab,kw

**#17** (Preeclamptic):ti,ab,kw

**#18** (maternal):ti,ab,kw OR (pregnancy):ti,ab,kw OR (pregnant):ti,ab,kw

**#19** (pre-eclampsia):ti,ab,kw OR (hypertension):ti,ab,kw OR (blood pressure):ti,ab,kw

**#20** #18 and #19

**#21** #1 or #2 or #3 or #4 or #5 or #6 or #7 or #8 or #9 or #10 or #11 or #12 or #13 or #14 or #15 or #16 or #17 or #20

**#22** MeSH descriptor: [Air Pollution] explode all trees

**#23** MeSH descriptor: [Particulate Matter] explode all trees

**#24** (Air Pollution):ti,ab,kw OR (Air Pollutions):ti,ab,kw OR (Pollution, Air):ti,ab,kw OR (Air Quality):ti,ab,kw OR (particulate matter 2.5):ti,ab,kw

**#25** (fine particulate matter):ti,ab,kw OR (inhalable fine particles):ti,ab,kw OR (inhalable particles PM2.5):ti,ab,kw OR (particulate matter 2.5 micrometers):ti,ab,kw OR (particulate matter 2.5):ti,ab,kw

**#26** (air pollution):ti,ab,kw OR (aerial pollution):ti,ab,kw OR (air contamination):ti,ab,kw OR (air pollutioning):ti,ab,kw OR (air-borne pollution):ti,ab,kw

**#27** (airborne pollution):ti,ab,kw OR (atmosphere pollution):ti,ab,kw OR (atmospheric pollution):ti,ab,kw OR (polluted air):ti,ab,kw OR (polluted atmosphere):ti,ab,kw

**#28** (pollution, air):ti,ab,kw OR (air pollution):ti,ab,kw OR (Air Pollutant):ti,ab,kw OR (air pollutants):ti,ab,kw OR (air pollutants, occupational):ti,ab,kw

**#29** (pollutant, air):ti,ab,kw OR (air pollutant):ti,ab,kw OR (particulate matter):ti,ab,kw OR (Air Pollutants):ti,ab,kw OR (Pollutants, Air):ti,ab,kw

**#30** (Air Pollutant):ti,ab,kw OR (Pollutant, Air):ti,ab,kw OR (Air Pollutants, Environmental):ti,ab,kw OR (Environmental Pollutants, Air):ti,ab,kw OR (Air Environmental Pollutants):ti,ab,kw

**#31** (Pollutants, Air Environmental):ti,ab,kw OR (Environmental Air Pollutants):ti,ab,kw OR (Pollutants, Environmental Air):ti,ab,kw OR (particulate matter):ti,ab,kw OR (Airborne Particulate Matter):ti,ab,kw

**#32** (Particulate Matter, Airborne):ti,ab,kw OR (Ambient Particulate Matter):ti,ab,kw OR (Particulate Matter, Ambient):ti,ab,kw OR (Air Pollutants, Particulate):ti,ab,kw OR (Particulate Air Pollutants):ti,ab,kw

**#33** (Ultrafine Particulate Matter):ti,ab,kw OR (Particulate Matter, Ultrafine):ti,ab,kw OR (Ultrafine Particle):ti,ab,kw OR (Particle, Ultrafine):ti,ab,kw OR (Ultrafine Particles):ti,ab,kw

**#34** (Particles, Ultrafine):ti,ab,kw OR (Ultrafine Fibers):ti,ab,kw OR (Ultrafine Fiber):ti,ab,kw OR (Fiber, Ultrafine):ti,ab,kw OR (traffic pollution):ti,ab,kw

**#35** (PM2.5):ti,ab,kw OR (outdoor pollution):ti,ab,kw OR (indoor pollution):ti,ab,kw

**#36** #22 or #23 or #24 or #25 or #26 or #27 or #28 or #29 or #30 or #31 or #32 or #33 or #34 or #35

**#37** #21 and #36

**Web of Science**

**#1** Hypertension, Pregnancy-Induced (Topic) OR Hypertension, Pregnancy Induced (Topic) OR Pregnancy-Induced Hypertension (Topic) OR Gestational Hypertension (Topic) OR Hypertension, Gestational (Topic) OR Pregnancy Induced Hypertension (Topic) OR Hypertensions, Pregnancy Induced (Topic) OR Induced Hypertension, Pregnancy (Topic) OR Induced Hypertensions, Pregnancy (Topic) OR Transient Hypertension, Pregnancy (Topic) OR Hypertension, Pregnancy Transient (Topic) OR Pregnancy Transient Hypertension (Topic) OR hypertensive disorders of pregnancy (Topic) OR HDP (Topic) OR maternal hypertension (Topic) OR hypertension during pregnancy (Topic) OR hypertension in pregnancy (Topic) OR hypertension induced by pregnancy (Topic) OR hypertension, maternal (Topic) OR hypertension, pregnancy induced (Topic) OR hypertension, pregnancy-induced (Topic) OR hypertensive disorder of pregnancy (Topic) OR PIH (Topic) OR pregnancy induced hypertension (Topic) OR pregnancy associated hypertension (Topic) OR pregnancy hypertension (Topic) OR pregnancy induced hypertension (Topic) OR pregnancy-induced hypertension (Topic) OR Pre-Eclampsia (Topic) OR Preeclampsia (Topic) OR pre eclampsia (Topic) OR pre-eclampsia (Topic) OR Preeclamptic (Topic) OR proteinuric hypertension of pregnancy (Topic) OR preeclampsia (Topic) OR Pre Eclampsia (Topic) OR Preeclampsia (Topic) OR Pregnancy Toxemias (Topic) OR Pregnancy Toxemia (Topic) OR Toxemia, Pregnancy (Topic) OR Edema-Proteinuria-Hypertension Gestosis (Topic) OR Edema Proteinuria Hypertension Gestosis (Topic) OR Gestosis, Edema-Proteinuria-Hypertension (Topic) OR Hypertension-Edema-Proteinuria Gestosis (Topic) OR Gestosis, Hypertension-Edema-Proteinuria (Topic) OR Hypertension Edema Proteinuria Gestosis (Topic) OR Proteinuria-Edema-Hypertension Gestosis (Topic) OR Gestosis, Proteinuria-Edema-Hypertension (Topic) OR Proteinuria Edema Hypertension Gestosis (Topic) OR EPH Complex (Topic) OR EPH Toxemias (Topic) OR EPH Toxemia (Topic) OR Toxemia, EPH (Topic) OR Toxemias, EPH (Topic) OR EPH Gestosis (Topic) OR Gestosis, EPH (Topic) OR Toxemias, Pregnancy (Topic) OR Toxemia Of Pregnancy (Topic) OR Of Pregnancies, Toxemia (Topic) OR Of Pregnancy, Toxemia (Topic) OR Pregnancies, Toxemia Of (Topic) OR Pregnancy, Toxemia Of (Topic) OR Toxemia Of Pregnancies (Topic) OR Preeclampsia Eclampsia 1 (Topic) OR 1, Preeclampsia Eclampsia (Topic) OR 1s, Preeclampsia Eclampsia (Topic) OR Eclampsia 1, Preeclampsia (Topic) OR Eclampsia 1s, Preeclampsia (Topic) OR Preeclampsia Eclampsia 1s (Topic) OR preeclampsia (Topic) OR Preeclamptic (Topic)

**#2** maternal (Topic) OR pregnancy (Topic) OR pregnant (Topic)

**#3** pre-eclampsia (Topic) OR hypertension (Topic) OR blood pressure (Topic)

**#4** #3 AND #2

**#5** #1 OR #4

**#6** Air Pollution (Topic) OR Air Pollutions (Topic) OR Pollution, Air (Topic) OR Air Quality (Topic) OR particulate matter 2.5 (Topic) OR fine particulate matter (Topic) OR inhalable fine particles (Topic) OR inhalable particles PM2.5 (Topic) OR particulate matter 2.5 micrometers (Topic) OR particulate matter 2.5 (Topic) OR air pollution (Topic) OR aerial pollution (Topic) OR air contamination (Topic) OR air pollutioning (Topic) OR air-borne pollution (Topic) OR airborne pollution (Topic) OR atmosphere pollution (Topic) OR atmospheric pollution (Topic) OR polluted air (Topic) OR polluted atmosphere (Topic) OR pollution, air (Topic) OR air pollution (Topic) OR Air Pollutant (Topic) OR air pollutants (Topic) OR air pollutants, occupational (Topic) OR pollutant, air (Topic) OR air pollutant (Topic) OR particulate matter (Topic) OR Air Pollutants (Topic) OR Pollutants, Air (Topic) OR Air Pollutant (Topic) OR Pollutant, Air (Topic) OR Air Pollutants, Environmental (Topic) OR Environmental Pollutants, Air (Topic) OR Air Environmental Pollutants (Topic) OR Pollutants, Air Environmental (Topic) OR Environmental Air Pollutants (Topic) OR Pollutants, Environmental Air (Topic) OR particulate matter (Topic) OR Airborne Particulate Matter (Topic) OR Particulate Matter, Airborne (Topic) OR Ambient Particulate Matter (Topic) OR Particulate Matter, Ambient (Topic) OR Air Pollutants, Particulate (Topic) OR Particulate Air Pollutants (Topic) OR Ultrafine Particulate Matter (Topic) OR Particulate Matter, Ultrafine (Topic) OR Ultrafine Particle (Topic) OR Particle, Ultrafine (Topic) OR Ultrafine Particles (Topic) OR Particles, Ultrafine (Topic) OR Ultrafine Fibers (Topic) OR Ultrafine Fiber (Topic) OR Fiber, Ultrafine (Topic) OR traffic pollution (Topic) OR PM2.5 (Topic) OR outdoor pollution (Topic) OR indoor pollution (Topic)

**#7** #5 AND #6

**Embase**

**#1** 'preeclampsia'/exp OR 'maternal hypertension'/exp

**#2** 'gestational hypertension':ab,ti OR 'hypertension, gestational':ab,ti OR 'hypertensions, pregnancy induced':ab,ti OR 'induced hypertension, pregnancy':ab,ti OR 'induced hypertensions, pregnancy':ab,ti OR 'transient hypertension, pregnancy':ab,ti OR 'hypertension, pregnancy transient':ab,ti OR 'pregnancy transient hypertension':ab,ti OR 'hypertensive disorders of pregnancy':ab,ti OR hdp:ab,ti OR 'maternal hypertension':ab,ti OR 'hypertension during pregnancy':ab,ti OR 'hypertension in pregnancy':ab,ti OR 'hypertension induced by pregnancy':ab,ti OR 'hypertension, maternal':ab,ti OR 'hypertension, pregnancy induced':ab,ti OR 'hypertension, pregnancy-induced':ab,ti OR 'hypertensive disorder of pregnancy':ab,ti OR pih:ab,ti OR 'pregnancy associated hypertension':ab,ti OR 'pregnancy hypertension':ab,ti OR 'pregnancy induced hypertension':ab,ti OR 'pregnancy-induced hypertension':ab,ti OR 'proteinuric hypertension of pregnancy':ab,ti OR 'pre eclampsia':ab,ti OR 'pregnancy toxemias':ab,ti OR 'pregnancy toxemia':ab,ti OR 'toxemia, pregnancy':ab,ti OR 'edema-proteinuria-hypertension gestosis':ab,ti OR 'edema proteinuria hypertension gestosis':ab,ti OR 'gestosis, edema-proteinuria-hypertension':ab,ti OR 'hypertension-edema-proteinuria gestosis':ab,ti OR 'gestosis, hypertension-edema-proteinuria':ab,ti OR 'hypertension edema proteinuria gestosis':ab,ti OR 'proteinuria-edema-hypertension gestosis':ab,ti OR 'gestosis, proteinuria-edema-hypertension':ab,ti OR 'proteinuria edema hypertension gestosis':ab,ti OR 'eph complex':ab,ti OR 'eph toxemias':ab,ti OR 'eph toxemia':ab,ti OR 'toxemia, eph':ab,ti OR 'toxemias, eph':ab,ti OR 'eph gestosis':ab,ti OR 'gestosis, eph':ab,ti OR 'toxemias, pregnancy':ab,ti OR 'toxemia of pregnancy':ab,ti OR 'of pregnancies, toxemia':ab,ti OR 'of pregnancy, toxemia':ab,ti OR 'pregnancies, toxemia of':ab,ti OR 'pregnancy, toxemia of':ab,ti OR 'toxemia of pregnancies':ab,ti OR 'preeclampsia eclampsia 1':ab,ti OR '1, preeclampsia eclampsia':ab,ti OR '1s, preeclampsia eclampsia':ab,ti OR 'eclampsia 1, preeclampsia':ab,ti OR 'eclampsia 1s, preeclampsia':ab,ti OR 'preeclampsia eclampsia 1s':ab,ti OR preeclampsia:ab,ti OR preeclamptic:ab,ti

**#3** maternal:ab,ti OR pregnancy:ab,ti OR pregnant:ab,ti

**#4** 'pre eclampsia':ab,ti OR hypertension:ab,ti OR 'blood pressure':ab,ti

**#5** #3 AND #4

**#6** #1 OR #2 OR #5

**#7** 'particulate matter 2.5'/exp OR 'air pollution'/exp OR 'air pollutant'/exp OR 'particulate matter'/exp

**#8** 'air pollutions':ab,ti OR 'air quality':ab,ti OR 'fine particulate matter':ab,ti OR 'inhalable fine particles':ab,ti OR 'inhalable particles pm2.5':ab,ti OR 'particulate matter 2.5 micrometers':ab,ti OR 'particulate matter 2.5':ab,ti OR 'aerial pollution':ab,ti OR 'air contamination':ab,ti OR 'air pollutioning':ab,ti OR 'air-borne pollution':ab,ti OR 'airborne pollution':ab,ti OR 'atmosphere pollution':ab,ti OR 'atmospheric pollution':ab,ti OR 'polluted air':ab,ti OR 'polluted atmosphere':ab,ti OR 'pollution, air':ab,ti OR 'air pollution':ab,ti OR 'air pollutants, occupational':ab,ti OR 'air pollutants':ab,ti OR 'pollutants, air':ab,ti OR 'air pollutant':ab,ti OR 'pollutant, air':ab,ti OR 'air pollutants, environmental':ab,ti OR 'environmental pollutants, air':ab,ti OR 'air environmental pollutants':ab,ti OR 'pollutants, air environmental':ab,ti OR 'environmental air pollutants':ab,ti OR 'pollutants, environmental air':ab,ti OR 'particulate matter':ab,ti OR 'airborne particulate matter':ab,ti OR 'particulate matter, airborne':ab,ti OR 'ambient particulate matter':ab,ti OR 'particulate matter, ambient':ab,ti OR 'air pollutants, particulate':ab,ti OR 'particulate air pollutants':ab,ti OR 'ultrafine particulate matter':ab,ti OR 'particulate matter, ultrafine':ab,ti OR 'ultrafine particle':ab,ti OR 'particle, ultrafine':ab,ti OR 'ultrafine particles':ab,ti OR 'particles, ultrafine':ab,ti OR 'ultrafine fibers':ab,ti OR 'ultrafine fiber':ab,ti OR 'fiber, ultrafine':ab,ti OR 'traffic pollution':ab,ti OR pm2.5:ab,ti OR 'outdoor pollution':ab,ti OR 'indoor pollution':ab,ti

**#9** #7 OR #8

**#10** #6 AND #9
